# S-Nitrosation of E3 Ubiquitin Ligase Complex Components Regulates Hormonal Signalings in Arabidopsis

**DOI:** 10.3389/fpls.2021.794582

**Published:** 2022-02-04

**Authors:** Maria Cecilia Terrile, Nuria Malena Tebez, Silvana Lorena Colman, Julieta Lisa Mateos, Esperanza Morato-López, Nuria Sánchez-López, Alicia Izquierdo-Álvarez, Anabel Marina, Luz Irina A. Calderón Villalobos, Mark Estelle, Antonio Martínez-Ruiz, Diego Fernando Fiol, Claudia Anahí Casalongué, María José Iglesias

**Affiliations:** ^1^Instituto de Investigaciones Biológicas, UE-CONICET-UNMDP, Facultad de Exactas y Naturales, Universidad Nacional de Mar del Plata, Mar del Plata, Argentina; ^2^Instituto de Fisiología, Biología Molecular y Neurociencias (IFIBYNE), CONICET-UBA, Buenos Aires, Argentina; ^3^Servicio de Proteómica, Centro de Biología Molecular “Severo Ochoa”, CSIC-UAM, Madrid, Spain; ^4^Unidad de Investigación, Hospital Universitario Santa Cristina, Instituto de Investigación Sanitaria Princesa (IIS-IP), Madrid, Spain; ^5^Molecular Signal Processing Department, Leibniz Institute of Plant Biochemistry (IPB), Halle (Saale), Germany; ^6^KWS Gateway Research Center, LLC., BRDG Park at The Danforth Plant Science Center, St. Louis, MO, United States; ^7^Section of Cell and Developmental Biology, University of California, San Diego, La Jolla, CA, United States

**Keywords:** *Arabidopsis thaliana*, auxin, jasmonates, SCF E3 ubiquitin ligase, S-nitrosation

## Abstract

E3 ubiquitin ligases mediate the last step of the ubiquitination pathway in the ubiquitin-proteasome system (UPS). By targeting transcriptional regulators for their turnover, E3s play a crucial role in every aspect of plant biology. In plants, SKP1/CULLIN1/F-BOX PROTEIN (SCF)-type E3 ubiquitin ligases are essential for the perception and signaling of several key hormones including auxins and jasmonates (JAs). F-box proteins, TRANSPORT INHIBITOR RESPONSE 1 (TIR1) and CORONATINE INSENSITIVE 1 (COI1), bind directly transcriptional repressors AUXIN/INDOLE-3-ACETIC ACID (AUX/IAA) and JASMONATE ZIM-DOMAIN (JAZ) in auxin- and JAs-depending manner, respectively, which permits the perception of the hormones and transcriptional activation of signaling pathways. Redox modification of proteins mainly by S-nitrosation of cysteines (Cys) residues *via* nitric oxide (NO) has emerged as a valued regulatory mechanism in physiological processes requiring its rapid and versatile integration. Previously, we demonstrated that TIR1 and *Arabidopsis thaliana* SKP1 (ASK1) are targets of S-nitrosation, and these NO-dependent posttranslational modifications enhance protein-protein interactions and positively regulate SCF^TIR1^ complex assembly and expression of auxin response genes. In this work, we confirmed S-nitrosation of Cys140 in TIR1, which was associated *in planta* to auxin-dependent developmental and stress-associated responses. In addition, we provide evidence on the modulation of the SCF^COI1^ complex by different S-nitrosation events. We demonstrated that S-nitrosation of ASK1 Cys118 enhanced ASK1-COI1 protein-protein interaction. Overexpression of non-nitrosable ask1 mutant protein impaired the activation of JA-responsive genes mediated by SCF^COI1^ illustrating the functional relevance of this redox-mediated regulation *in planta*. *In silico* analysis positions COI1 as a promising S-nitrosation target, and demonstrated that plants treated with methyl JA (MeJA) or S-nitrosocysteine (NO-Cys, S-nitrosation agent) develop shared responses at a genome-wide level. The regulation of SCF components involved in hormonal perception by S-nitrosation may represent a key strategy to determine the precise time and site-dependent activation of each hormonal signaling pathway and highlights NO as a pivotal molecular player in these scenarios.

## Introduction

In eukaryotic organisms, the ubiquitin-proteasome system (UPS) participates in the selective degradation of proteins modulating, in turn, a wide variety of physiological processes (Hershko and Ciechanover, 1998). Substrate proteins are ubiquitinated through the sequential action of E1 (ubiquitin-activating enzymes), E2 (ubiquitin-conjugating enzymes), and E3 (ubiquitin ligases) prior to their degradation by the 26S proteasome (Hershko and Ciechanover, 1998). Genome-wide analyses of UPS components indicate that plants encode a significantly higher number of E3 ligases compared to E1s and E2s and E3s in other kingdoms (Vierstra, 2009; Iglesias et al., 2021). Diversification and complexity of UPS regulation in plants could be a consequence of its sessile condition. To adapt plant growth and development to dynamically changing environments, several signaling pathways rely on posttranslational regulation, including phosphorylation, acetylation, hydroxylation, or ubiquitination for rapid protein degradation (Spoel, 2018; Vu et al., 2018). In plants, SKP1/CULLIN1/F-BOX PROTEIN (SCF)-type E3 ubiquitin ligases constitute the best characterized multimeric cullin-RING E3-type ligase. SCFs primarily consist of four subunits: CULLIN1 (CUL1), S-PHASE KINASE-ASSOCIATED PROTEIN 1 (SKP1, or ASK1 in *Arabidopsis thaliana*), RING-BOX PROTEIN 1 (RBX1), and a substrate receptor F-box protein (FBP). CUL1 functions as a scaffold protein, while RBX1 serves as a docking site for the ubiquitin-loaded E2, and an ASK1-FBP dimer provides specificity to the SCF-type E3 by recognizing directly the ubiquitination substrate (Hua and Vierstra, 2011). The UPS is central to hormonal regulation in plants since protein ubiquitination and proteasome degradation serve for the orchestration of hormone homeostasis, transport, and perception (Kelley and Estelle, 2012; Dharmasiri et al., 2013). In particular, the activation of auxin and jasmonates (JAs) signaling pathways involves the degradation of transcriptional repressors through the action of SCF E3 ubiquitin ligases. TRANSPORT INHIBITOR RESPONSE 1 (TIR1)/AUXIN-RELATED F-BOX (AFBs) and CORONATINE INSENSITIVE 1 (COI1) together with their ubiquitination substrates AUXIN/INDOLE-3-ACETIC ACID (AUX/IAA) and JASMONATE ZIM-DOMAIN (JAZ), constitute the co-receptor systems, respectively (Calderón Villalobos et al., 2012). Upon hormone binding by SCF^TIR1/AFBs^-AUX/IAA and SCF^COI1^-JAZs co-receptor complexes, poly-ubiquitination and degradation of AUX/IAA and JAZ transcriptional repressors take place. This consecutively promotes shuffling of the transcriptional machinery promoting auxin- and JAs-responsive genes activation.

Nitric oxide (NO) is a hydrophobic, free radical, and highly diffusible short-lived gas involved in a broad spectrum of regulatory functions in prokaryotes and eukaryotes. In plants, NO regulates a large number of physiological processes mediated by different hormones (Baudouin and Hancock, 2014). The redox modification of proteins, mainly by S-nitrosation of cysteines (Cys) *via* NO has emerged as a valued regulatory mechanism in physiological processes that require rapid and versatile integration (Paris et al., 2013; Gupta et al., 2020). S-nitrosation consists of the formation of a nitrosothiol (SNO) through the incorporation of a NO moiety into a reactive thiol group of a Cys residue. This modification has been associated with the modulation of a protein’s subcellular localization, stability, interactions with proteins and DNA, and biochemical activities (Astier and Lindermayr, 2012).

NO sensing is associated with UPS-mediated protein degradation that impacts plant developmental processes and stress responses in plants (León and Costa-Broseta, 2020). NO is partially sensed through modulation of UPS-mediated proteolysis by posttranslational modifications (e.g. S-nitrosation) or through targeted degradation by the N-degron pathway (Iglesias et al., 2021). NO sensing executed by S-nitrosation regulates protein degradation directly (targeting central components of UPS machinery), or indirectly, modifying protein-protein interactions, localization, or enzyme activities with consequences on protein degradation (Rizza et al., 2014).

We have previously demonstrated that TIR1 and ASK1 subunits of the SCF^TIR1^ E3 ubiquitin ligase complex are targeted by redox posttranslational modifications (Terrile et al., 2012; Iglesias et al., 2018). S-nitrosation of TIR1 and ASK1 promotes SCF^TIR1^ complex assembly increasing degradation of AUX/IAA transcriptional repressors and activation of auxin-mediated gene expression (Terrile et al., 2012; Iglesias et al., 2018). Therefore, NO has been postulated as an extra player in the modulation of auxin signaling in *A. thaliana* (Wang and Estelle, 2014; Correa-Aragunde et al., 2016). Since ASK1 can interchangeably associate with FBP proteins of different SCF complexes involved in diverse signaling pathways, the study of the regulation of SCF assembly is of high relevance. Considering the similarities between auxin and JA signaling pathways, it is plausible that NO-mediated S-nitrosation of SCF components impacts auxin and also JAs sensing and regulation. In this work, we give new insights on the impact of TIR1 S-nitrosation in auxin responses and explore the NO modulation of the SCF^COI1^ E3 ubiquitin ligase complex by multiple S-nitrosation events, with consequent effects on the activation of the JAs signaling pathway.

## Materials and Methods

### Plant Material and Growth Conditions

*Arabidopsis thaliana* transgenic lines *tir1-1* 35S:tir1 C140A in Col-0 ecotype have been previously described (Terrile et al., 2012). The seeds were surface-sterilized and stratified at 4°C in the dark for 2–4 days. Then, the seeds were plated on *A. thaliana* salts (ATS) medium (Wilson et al., 1990) containing 1% sucrose with 0.8% agar or transferred to soil and grown at 23°C under 120 μmol photons m^2^ sec^–1^ with 16 h:8 h light:dark cycles.

*Nicotiana benthamiana* L. plants were grown in soil [3 parts of Grow Mix Multipro (Terrafertil, Argentina) with 1 part of vermiculite] with a 16-h photoperiod (150 μE m^2^ s^–1^ of photosynthetically active radiation) at 25 °C and 60% relative humidity in a greenhouse.

For transient expression of ASK1 protein, leaves from 4-week-old *N. benthamiana* were infiltrated with *Agrobacterium tumefaciens* strain GV3101 carrying 35S:ASK1 pEarleyGate203 expression vector (or 35S:ask1 C37A, 35S:ask1 C59A, 35S:ask1 C118A) together with p19 (a gene silencing suppressor) as described in Iglesias et al. (2018). Infiltration solution (10 mM MgCl_2_, 10 mM MES pH 5.6, 100 μM acetosyringone) was used as control. Infiltrated leaves were harvested 24 h post-infiltration and stored at −80 °C.

### Salt Stress Treatment

Ten-day-old seedlings grown on ATS agar medium were transferred into liquid ATS medium supplemented with 200 mM NaCl during 3 d, as high salt stress, and then chlorophyll was extracted with 80% acetone for 30 min in the dark. The amounts of chlorophyll were measured spectrophotometrically at 645 and 663 nm (Arnon, 1949). Three independent experiments (*n* = 150 seedlings) were performed. The working NaCl concentration corresponds to the condition that promotes a 50% reduction in chlorophyll content in Col-0 plants (Iglesias et al., 2010).

### S-Nitrosocysteine Preparation

S-nitrosocysteine were prepared as previously described by Izquierdo-Álvarez et al. (2018). Briefly, 1 ml of 200 mM L-Cys in 1 M HCl was mixed with 1 ml of 200 mM NaNO_2_ for 30 min in darkness at room temperature. Then, 2 ml of 1 M potassium phosphate buffer, pH 7.4, was added. The NO-Cys concentration was determined spectrophotometrically (extinction coefficient; ε338 = 900 M^–1^ cm^–1^) and aliquots were stored at –80°C.

### Pull-Down Assays

The *in vitro* pull-down assay with TIR1/tir1 C140A -myc proteins synthesized by the TNT-coupled wheat germ extract system (Promega) was described previously (Parry et al., 2009). The synthesis of recombinant glutathione S-transferase (GST)-ASK1 or GST-ask1 C118A proteins was described in Iglesias et al. (2018). Briefly, GST-ASK1 or GST-ask1 C118A proteins were expressed in *Escherichia coli* and then purified with glutathione-sepharose beads (GE Healthcare, United States; Iglesias et al., 2018). Five μg of GST-ASK1 or GST-ask1 C118A purified proteins were immobilized and incubated with equal amounts of TNT-synthetized TIR1-myc or tir1-myc C140A (5–10 μl of protein extract) in the presence of 100 μM NO-Cys in 200 μl HEN buffer (25 mM HEPES pH 7.7, 1 mM EDTA, 0.1 mM neocuproine -Sigma-Aldrich, United States) in darkness for 1 h. Samples were washed with HENS (HEN with 2% SDS) and eluted in 50 mM Tris-HCl pH 8.0 supplemented with 200 mM NaCl and 10 mM glutathione (GSH). TIR1/tir1-myc C140A eluted proteins were detected by immunoblotting. Proteins were run on SDS-PAGE, blotted into nitrocellulose membranes, incubated with primary anti-myc antibody (Sigma-Aldrich, United States) overnight, and then with secondary antibody coupled to peroxidase (Invitrogen, United States) for 2 h, and finally visualized using the enhanced chemiluminescence (ECL) kit (Amersham Biosciences).

### Yeast Two-Hybrid Analysis

*Saccharomyces cerevisiae* strain EGY48 [pSH18-34] (Clontech, United States; Gietz et al., 1992) were used to co-express DBD-COI1 and AD-ASK1 (or AD-mutated ask1). Yeasts were grown on SD–U–H–T selective media supplemented with 100 μM sodium nitroprusside (SNP) and 5-bromo-4-chloro-indolyl-b-D-galactopyranoside (X-Gal) to develop β-galactosidase activity under ambient light for 48 h as described in Iglesias et al. (2018).

### Mass Spectrometry Analysis of Transport Inhibitor Response 1 S-Nitrosation by Reverse Phase-Liquid Chromatography Tandem Mass Spectrometry (RP-LC-MS/MS)

GST-TIR1 was expressed in SF9 insect cells and purified by glutathione affinity chromatography as previously published (Tan et al., 2007; Calderón Villalobos et al., 2012). Fractions were buffer exchanged to 15% glycerol containing buffer and stored at –80 °C until use. GST-TIR1 was S-nitrosated with 1 mM S-nitrosoglutathione (GSNO) in HENS buffer for 30 min in the dark. Then proteins were subjected to a modification of the biotin switch assay using either dithiothreitol (DTT) (redox switch) or ascorbate (nitrosothiol switch) as reducing agent and iodoacetamide (IAM) for labeling, thus switching reversibly oxidized or S-nitrosated Cys to carbamidomethylated Cys (Izquierdo-Álvarez et al., 2018). Free Cys were blocked with N-ethylmaleimide (NEM, Sigma, United States) in 4 volumes of blocking buffer (225 mM HEPES pH 7.2, 0.9 mM EDTA, 90 μM neocuproine, 2.5% SDS, and 30 mM NEM) for 30 min at 37°C. For the redox switch, protein was precipitated with acetone, resuspended in HENS containing 1 mM DTT, and incubated for 10 min at 15°C. Then, it was precipitated with acetone and resuspended in HENS containing IAM (Sigma, United States), and incubated for 1 h at 25°C. For the nitrosothiol switch, protein was precipitated with acetone and resuspended in HENS containing 100 mM ascorbate and IAM for 1 h at 25°C. Proteins were run in SDS-PAGE and gel bands were digested as fully described in Iglesias et al. (2018).

Protein identification by LC-MS/MS and identification of posttranslational modifications were carried out in the Centro de Biología Molecular Severo Ochoa (CBMSO, Madrid, Spain) protein chemistry facility, a member of ProteoRed network. The desalted protein digest was dried, resuspended in 10 μl of 0.1% formic acid, and analyzed by RP-LC-MS/MS in an Easy-nLC II system coupled to an ion trap LTQ-Orbitrap-Velos-Pro mass spectrometer (Thermo Fisher Scientific, United States). The peptides were concentrated (online) by reverse phase chromatography using a 0.1 mm × 20 mm precolumn Acclaim PepMap C18, 5 μm, 100 A (Thermo Fisher Scientific, United States), and then separated using a 0.075 mm × 100 mm column Acclaim PepMap C18, 3 μm, 100 A (Thermo Fisher Scientific, United States) operating at 0.3 μl/min. Peptides were eluted using a 60-min gradient from 5 to 40% solvent B (solvent A: 0,1% formic acid in water; solvent B: 0,1% formic acid, 80% acetonitrile -ACN- in water). Electrospray ionization (ESI) was done using a Nano-bore emitter stainless steel ID 30 μm interface. The Orbitrap resolution was set at 30,000. The mass spectrometer was operated in the selected MS/MS ion monitoring mode (SMIM mode). In this mode, the LTQ-Orbitrap-Velos-Pro detector was programmed to perform, along the same entire gradient, a continuous sequential operation in the MS/MS mode on the doubly or triply charged ions corresponding to the peptide/s selected previously from the theoretical prediction. Peptide identification from raw data was carried out using PEAKS Studio 6 search engine (Bioinformatics Solutions Inc., Waterloo, Ontario, Canada).

### RNA Isolation and Quantitative Real-Time RT-qPCR

Total RNA from *N. benthamiana* infiltrated leaves was extracted using TRIzol reagent (Invitrogen, United States) according to the manufacturer’s recommendations. Samples were incubated with RQ1 RNase-free DNase (Promega, United States) to remove DNA contamination. cDNAs were synthesized from 1 μg of total RNAs using IMPROM II (Thermo Fisher Scientific, United States) with random primers (Biodynamics SRL, Argentine). The expression of a subset of JA response genes (NbVSP1-Niben101Scf34114g00003; NbMYC2-Niben101Scf06822g04004.1; NbASA1-Niben101Scf06493g00022.1; NbPR4-X60281) was analyzed by qPCR. The primers used are listed in Supplementary Table 1. qPCR reactions were performed in triplicates (40 cycles at 95 °C for 10 min and 1 min at 60 °C) in a Step One real-time PCR system (Applied Biosystems, United States) using SYBR green PCR master mix (Applied Biosystems, United States). EF-1α was used as a house keeping gene (Schmidt and Delaney, 2010). The comparative cycle threshold (Ct) method was used to determine relative expression levels (Schmittgen and Livak, 2008). Three independent experiments involving 2 plants per treatment with similar ASK1 overexpression were performed.

### Prediction of S-Nitrosation Sites

Five different software were used to predict S-nitrosation sites: GPS-SNO 1.0 (Xue et al., 2010),^1^ iSNO-PseAAC (Xu et al., 2013a),^2^ iSNO-AAPair (Xu et al., 2013b),^3^ DeepNitro (Xie et al., 2018),^4^ and pCysMod (Li et al., 2021).^5^

### Protein Modeling

Cys118 S-nitrosation of ASK1 was modeled in PyMol (The PyMOL Molecular Graphics System, Version 2.0 Schrödinger, LLC.) using the Modeler program (Eswar et al., 2006) included in the PyMod plugin (Janson and Paiardini, 2021). Cys118 was replaced in ASK1 chains of the structure of the COI1-ASK1 complex (PDB ID: 3OGL) using Vienna-PTM 2.0 (Margreitter et al., 2013) and the resulting structure was submitted to energy minimization to reduce the global internal energy of the system using Gromos (Reif et al., 2013). The surface electrostatic potential of structures was calculated in PyMOL using the Adaptive Poisson-Boltzmann Solver (APBS) tool (Jurrus et al., 2018).

### Transcriptomic Analysis

For comparison of transcriptomic effects mediated by NO and JA, we used RNA-Seq public data. For the analyses of RNA-seq data from treatment, raw reads from GSE81361 (Hussain et al., 2016) were downloaded from National Center for Biotechnology Information (NCBI). Reads were mapped to the Arabidopsis genome (TAIR10) using a TopHat-Bowtie pipeline (Trapnell et al., 2009) with default parameters except for maximum intron length set at 5,000 nucleotides. Differential gene expression analysis was done using R with the ASpli package (Mancini et al., 2021). Genes with | logFC| > 1 and *p*-value < 0.0001 were considered as differentially expressed between NO-Cys and mock. To obtain transcriptional effects of JA, we selected genes that were found to respond to MeJA treatment in Hickman et al. (2017). The data on Hickman et al. (2017) corresponds to a time series of MeJA treated plants at 15 different time points. To have MeJA and NO-Cys comparable datasets and better define the effects of both treatments, in the time series experiment we used only differentially expressed genes (DEGs) after 6 h of MeJA incubation. DEGs under both treatments (NO and MeJA) were compared and common genes (2,106) were selected for downstream analysis. The logFC values for each gene in each experiment were calculated and used to evaluate the correlation. Values were represented in a scatter plot.

For gene ontology (GO), term analysis lists of genes of interest were submitted to DAVID Bioformatic Resources (Huang et al., 2009a,b). Only GO terms with at least 4 genes per GO term category and false discovery rate (FDR) < 0.01 were considered for downstream analysis.

### Statistical Analysis

The values shown in each figure are mean ± SE. The data were subjected to *t*-test or ANOVA (one-way ANOVA) and *post hoc* comparisons with Dunnet test (*p < 0.05, ^**^p < 0.01) using Graphpad Prism version 5.01 software.

## Results and Discussion

### S-Nitrosation of the F-Box Protein Transport Inhibitor Response 1 in SCF^TIR1^ Complex: Functional Relevance

**FIGURE 1:**
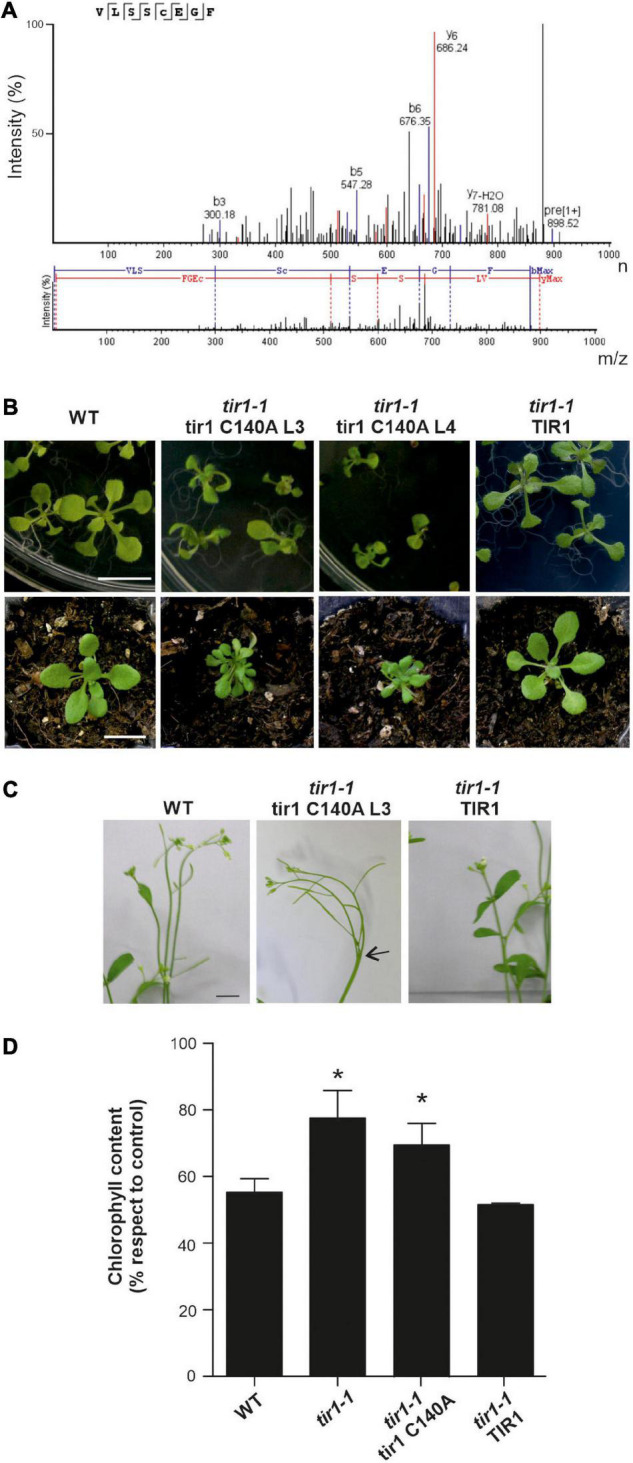
TIR1 S-nitrosation of Cys140 residue. **(A)** MS/MS spectrum showing fragmentation patterns that correspond with ions of the y (blue) and the b (red) series of S-carbamidomethylated Cys140 peptide after treatment of TIR1 with GSNO and ascorbate switch, which specifically substitutes S-nitrosated Cys to carbamidomethylation. **(B)** Phenotype of different *tir1-1* 35S:tir1 C140A transgenic lines overexpressing non-nitrosable TIR1 protein. Representative pictures of WT, *tir1-1* 35S:TIR1 and *tir1-1* 35S:tir1 C140A (L3 and L4) seedlings grown during 15 days on horizontally oriented *Arabidopsis thaliana* salts (ATS) agar plates (upper panels) and in soil during 21 days (lower panels) are shown. Scale bar: 1 cm. **(C)** A representative picture of morphological defects in inflorescences is shown. Arrow indicates fused bolts. Scale bar: 5 mm. **(D)** Ten-day-old seedlings were transferred to liquid ATS medium supplemented with 200 mM NaCl for 3 d. Chlorophyll content was measured spectrophotometrically and relativized to untreated seedlings (control). Data are mean values (± SE) of three independent experiments. *P < 0.05 (*t*-test against WT).

We have previously demonstrated NO-mediated TIR1 S-nitrosation by using a biotin switch assay and predicted two putative Cys, Cys140 and Cys480 (Terrile et al., 2012). Preliminary findings on TIR1 Cys140 S-nitrosation were confirmed in this work by MS. For that, TIR1 was obtained from insect cells, and treated with GSNO, a nitrosothiol that can S-nitrosate Cys residues. We first detected reversibly oxidized Cys residues in GSNO-treated TIR1 by a redox switch (Izquierdo-Álvarez et al., 2018) identifying the peptide including carbamidomethylated Cys140 (Supplementary Figure 1). To confirm Cys140 S-nitrosation, we perform a similar switch using ascorbate as a reducing agent. We detected by MS the same peptide with carbamidomethylated Cys140, indicative of S-nitrosation (Figure 1A). We were not able to detect the corresponding modification in Cys480 by MS, even though it was proposed as a second candidate of S-nitrosation (Terrile et al., 2012). At biochemical and physiological levels, mutation of TIR1 Cys480 residue had a mild effect on protein functionality compared with Cys140 (Terrile et al., 2012). There are multiple examples of proteins that suffer NO modifications in more than one Cys residue (Holtgrefe et al., 2008; Lindermayr et al., 2010; Hu et al., 2017; Iglesias et al., 2018). However, there is still no evidence of alternative S-nitrosation events associated with different NO intracellular levels, protein localization, or specific biological processes. In the case of ASK1, where two Cys residues are S-nitrosation targets, we found different strengths of S-nitrosation being Cys37 more susceptible to oxidation than Cys118 at low NO levels (Iglesias et al., 2018). Therefore, we do not discard additional S-nitrosation residues in TIR1. TIR1 Cys140 constituted a critical residue for TIR1–AUX/IAA interaction and TIR1 homodimerization, and consequently, transgenic plants expressing tir1 C140A or C140S presented impaired root physiological responses to IAA (Terrile et al., 2012; Dezfulian et al., 2016). In this study, we explore if S-nitrosation of TIR1 in Cys140 is implicated in other auxin-related phenotypes. Multiple mutants in TIR1/AFBs auxin receptors show defects in rosettes and inflorescences (Dharmasiri et al., 2005; Prigge et al., 2020). Similarly, *tir1-1* 35S:tir1 C140A plants exhibit small highly curled leaves and abnormal inflorescence branching while *tir1-1 35S:TIR1* plants do not show defects in organ development (Figures 1B,C). Since *tir1-1* mutants are not strongly affected in these phenotypes, it could be possible that the overexpression of a not-fully functional tir1 C140A version could be generating a negative dominant effect on other auxin receptor members. Regarding TIR1 role in the adaptive response of plants to environmental changes, it has been described that auxin signaling is downregulated under several stresses, and therefore *tir1-1* mutants showed increased tolerance to adverse conditions (Navarro et al., 2006; Iglesias et al., 2014). To analyze if S-nitrosation of TIR1 Cys140 is also implicated in stress responses, we evaluated *tir1-1* 35S:tir1 C140A seedling tolerance to salinity. Overexpression of non-nitrosated tir1 C140A protein did not complement *tir1-1* response in terms of chlorosis when exposed to high salinity treatments (200 mM NaCl) as TIR1 did (Figure 1D), suggesting that regulation of Cys140 residue by NO is relevant for diverse physiological responses in *A. thaliana*.

**FIGURE 2:**
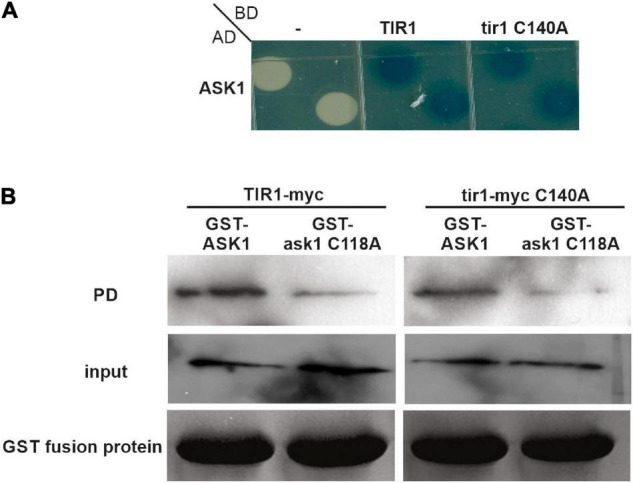
S-nitrosation of TIR1 does not affect TIR1-ASK1 protein-protein interaction. **(A)** Yeasts were co-transformed with the indicated constructs and grown on SD–U–H–T selective media and X-Gal to develop β-galactosidase activity. **(B)** TIR1-myc or tir1-myc C140A proteins were synthesized *in vitro* and incubated with recombinant GST-ASK1 or GST-ask1 C118A in the presence or absence of 0.1 mM NO-Cys as S-nitrosating agent. After pull-down (PD) reactions, recovery of the TIR1 and tir1-myc C140A proteins was assessed using anti-myc antibody (top panels). The middle panel shows the input of *in vitro* synthesized TIR1 and tir1-myc C140A in the PD reactions, whereas lower panels show the coomassie staining of the entire PD reactions as loading control for GST-ASK1 or GST-ask1 C118A proteins.

ASK1 scaffold protein of SCF complexes is S-nitrosated in Cys37 and Cys118 (Iglesias et al., 2018). Particularly, Cys118 lies on the SCF^TIR1^ complex in the interface between ASK1 and TIR1 and S-nitrosation of Cys118 in ASK1 reinforced the interaction of ASK1 with TIR1 (Iglesias et al., 2018). Since both TIR1 and ASK1 are targets of S-nitrosation, we decided to evaluate if TIR1 S-nitrosation in Cys140 also contributes to TIR1-ASK1 interaction as S-nitrosation of ASK1 Cys118 does, by yeast two-hybrid and pull-down assays. Yeast cells were co-transformed with AD-ASK1 and BD-TIR1 or BD-tir1 C140A and tested for β-Galactosidase reporter activity. Figure 2A shows no differences in the interaction between both pairs of proteins. For pull-down assays, GST-ASK1 was immobilized in GSH-sepharose beads and incubated with *in vitro*-translated TIR1-myc or tir1-myc C140A in the presence of 100 μM NO-Cys as an S-nitrosating agent. There were no differences between the amount of pull-down TIR1 and tir1 C140A proteins (Figure 2B), suggesting that TIR1 S-nitrosation in Cys140 is not required for ASK1-TIR1 interaction. In addition, the mutation in ask1 Cys118 causes a reduction in protein-protein interaction with both WT and tir1 C140A proteins. Pull-downs performed with GST protein as control, show that ASK1-TIR1 interaction is specific (Supplementary Figure 2).

### S-Nitrosation of ASK1 Scaffold Protein Participates in SCF^COI1^ Complex Regulation

**FIGURE 3:**
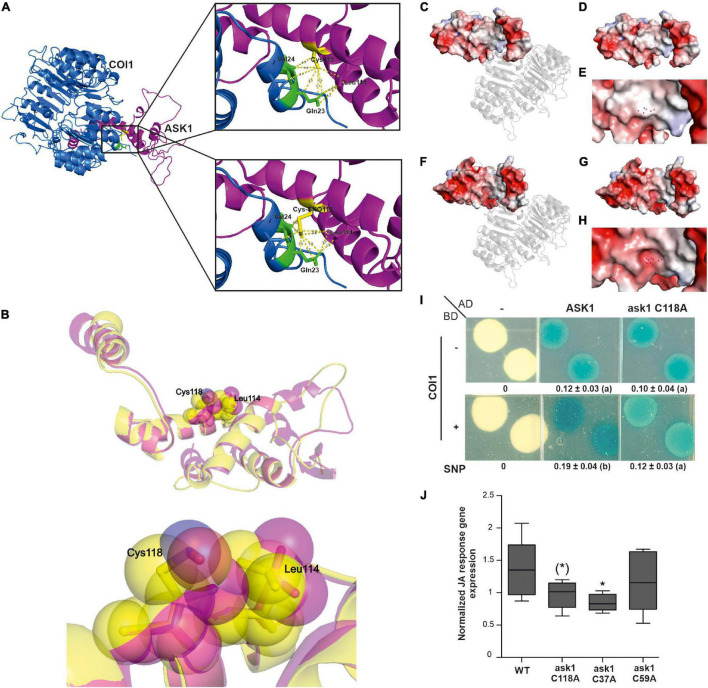
The Cys118 residue of ASK1 has a pivotal role in NO-mediated interaction with COI1. **(A)** Cartoon representations of the crystal structure of ASK1 and COI1 (upper image) and modeled structure of SNO-Cys118 ASK1 and COI1 (lower image). Zoom views show amino acids of COI1 interacting with Cys118 ASK1 and SNO-Cys118 ASK1, respectively **(B)** Superimposed cartoon representations of ASK1 crystal structure and SNO-Cys118 ASK1 modeled structure. Semitransparent Van der Walls spheres showing the conformational changes of Cys118 and Leu114 are depicted in the magnified image. Surface electrostatic potential of ASK1 illustrated with a cartoon representation of COI1 in the ASK1-COI1 complex **(C)** or alone **(D)** and a zoomed view of the area interacting with COI1 **(E).** Atoms of residue Cys118 are depicted as red points. Surface electrostatic potential of modeled NO-Cys118 ASK1 illustrated with a cartoon representation of COI1 in the ASK1-COI1 complex **(F)** or alone **(G)** and a zoomed view of the area interacting with COI1 **(H)**. Atoms of residue SNO-Cys118 are depicted as red points. The electrostatic potential is represented in a gradient from blue (electropositive) to red (electronegative). **(I)** Yeast two-hybrid assays were carried out to test the interaction between ASK1 or ask1 C118A and COI1 on SD–U–H–T selective media plus the addition of 100 μM SNP and X-Gal to develop β-galactosidase activity. Densitometry of yeast two-hybrid activity (*n* = 3; different letters indicate a significant difference at *P* ≤ 0.05, one-way ANOVA, *post hoc* Tukey). **(J)** Averaged change in expression level relative to EF1 (NbEF-1α-Niben101Scf12941g01003.1) for the subset of JA response genes. ASK1 WT or its mutants in Cys37, Cys59, and Cys118 were transiently expressed in leaves of 4-week-old *N. benthamiana* plants, and the expression of a subset of JA response genes (NbVSP1-Niben101Scf34114g00003; NbMYC2-Niben101Scf06822g04004.1; NbASA1-Niben101Scf06493g00022.1; NbPR4-X60281) was analyzed by qPCR 24 h after infiltration in 3 independent experiments. EF1 was used as housekeeping gene. Data were normalized to the median of each experiment for all the JA response genes. Box-plots (median, 1–3 interquartile range and 95% CI) of normalized expression. The significance of the effect of Cys mutation is indicated [*t*-test compared to ASK1 *p < 0.05, (*) *p* < 0.06].

COI1 is the closest FBP homolog to TIR1/AFBs auxin co-receptors in *A. thaliana*, serving as a substrate of JAZ proteins in the SCF^COI1^ complex for the perception and activation of JA responses (Parry et al., 2009; Sheard et al., 2010). Phylogenetic analysis indicated that both auxin and JA FBPs had one common ancestor in charophyte green algae, with no hormone-binding properties, and which developed into both receptors by duplication in the ancestor of land plants (Mutte et al., 2018). Therefore, although the amino acid residues associated with auxins and JAs perception are not conserved between both co-receptors, their primary sequences and protein structures present a high degree of similarity that could allow similar regulations (Supplementary Figure 3). Therefore, we decided to explore if ASK1 regulation by S-nitrosation in Cys37 and Cys118 could also impact SCF^COI1^ assembly. We analyzed the crystal structures of SCF^TIR1^ and SCF^COI1^ looking for similarities in the interfaces of S-nitrosated ASK1 with TIR1 and COI1, respectively. When analyzing TIR1 and COI1 amino acids in the proximity of ASK1 Cys118, a conserved Val residue with similar distances of interaction was found in both TIR1 (Val17) and COI1 (Val24) proteins (Supplementary Figure 4 and Figure 3A). Gln23 in COI1 and His16 in TIR1 are also surrounded by ASK1 Cys118. The modeling of ASK1 S-nitrosation with the addition of a nitrosothiol (-SNO) group to Cys118 (Cys-SNO) predicts a conformational change affecting the distance to Val24 and Gln23 of COI1 in 1.4 and 1.3 Å, respectively (Figures 3A,B). In addition, Leu114 of ASK1 is also shifted to the proximity of Cys118 reducing the distance to Gln23 from 3.5 to 2.9 Å (Figures 3A,B). Analysis of the electrostatic potential surface in the contact area of COI1-ASK1 when Cys118 is S-nitrosated (Cys-SNO) compared with unmodified Cys118 residue showed a significant change to a more electronegative surrounded (Figures 3C–H). This change in electrostatic potential values could alter the binding to COI1 since charge complementarity between ASK1 and COI1 results important in the stabilization of the complex. The *in silico* analysis of structural data suggested that S-nitrosation of ASK1 in Cys118 could be affecting its interaction with COI1. Therefore, we tested this hypothesis by evaluating the ASK1-COI1 interaction in yeast. Cells co-expressing AD-ASK1 and BD-COI1 were grown in the presence and absence of the slow-release NO donor, SNP. β-Galactosidase reporter expression shows that NO treatment enhanced ASK1-COI1 interaction (Figure 3I). To evaluate whether S-nitrosation of ASK1 in Cys 118 was particularly implicated in NO regulation of this interaction, we performed yeast two-hybrid assays with ask1 C118A mutant impaired in S-nitrosation (Iglesias et al., 2018). Compared to ASK1, ask1 C118A-COI1 protein-protein interaction was not increased by SNP treatment, showing that NO has a specific action when S-nitrosated Cys118 residue is present. ASK1 S-nitrosation at Cys118 might favor COI1-ASK1 assembly (Figure 3I). It has been demonstrated that the COI1 protein level is strictly regulated by a dynamic equilibrium of stabilization mediated by their assembly to the SCF complex and degradation *via* the 26S proteasome (Yan et al., 2013). COI1 is stabilized when COI1-ASK1 heterodimers are formed, which are then further stabilized by CUL1-ASK1 interaction in SCF^COI1^ complex assembly, and therefore protected from UPS degradation. S-nitrosation of ASK1 Cys118 and Cys37 enhanced COI1-ASK1 and ASK1-CUL1 interactions, respectively (Figure 3I; Iglesias et al., 2018). Thus, NO through two S-nitrosation events in ASK1 protein could be involved in the regulation of COI1 balance and part of the fine-tuning regulation of the JA signaling pathway. Strict regulation of SCF^COI1^complex assembly has been proposed for the activation of the JA signaling pathway required for developmental processes and defense responses in *A. thaliana* plants (Yan et al., 2013). To explore this hypothesis, the functional relevance of ASK1 S-nitrosation in the activation of JA signaling was analyzed *in planta*. ASK1 and non-nitrosable versions (ask1 C37A and ask1 C118A) were transiently expressed in *N. benthamiana* leaves, and JAs-responsive genes were analyzed by qPCR. A construct expressing a mutant in Cys59 of ASK1 (ask1 C59A), which is not a target of NO regulation and has no impact on SCF complex assembly (Iglesias et al., 2018), was used as control. Overexpression of ask1 C37A and ask1 C118A presented a reduced ability to induce JAs-regulated transcripts compared to ASK1 or ask1 C59A (Figure 3J), indicating the functional relevance of ASK1 Cys37 and Cys118 S-nitrosation on JA signaling regulation.

### Coronatine Insensitive 1 F-Box Protein as a Putative Target of S-Nitrosation

**FIGURE 4:**
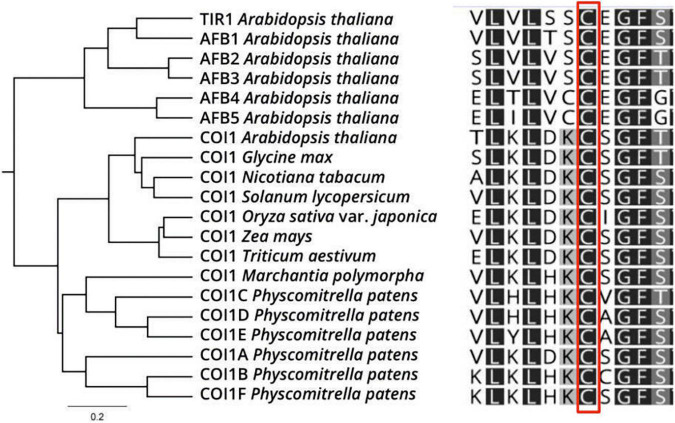
COI1 as a putative S-nitrosation target. COI1 orthologs of select plant species are aligned, showing the conservation in Cys148. Color code: dark gray: 100% similarity; medium gray: 80–100% similarity; light gray: 60–80% similarity; white: less than 60% similar.

Due to COI1 and TIR1 similarities at protein structure and sensing mechanism by SCF complexes in JAs and auxin signaling pathways, we decided to explore if the COI1 co-receptor could be a putative target of S-nitrosation by *in silico* analysis. We analyzed *A. thaliana* TIR1/AFBs and COI1 amino acid sequences from various plant species to determine the conservation of S-nitrosable TIR1 Cys140 residue in COI1 homologs. Protein sequence alignment showed a high degree of conservation of Cys residue at position 148 in AtCOI1 (corresponding to Cys140 in AtTIR1, Figure 4). However, AtCOI1 Cys487 (Cys480 in AtTIR1) presented a lower degree of conservation (Supplementary Figure 5). We used different S-nitrosation site-prediction algorithms to analyze the potential Cys targets of S-nitrosation in COI1 protein (GPS-SNO 1.0, iSNO-PseAAC, iSNO-AAPair, DeepNitro, and pCysMod software; Zhao et al., 2021). As a positive control, we included in the analysis the prediction of AtTIR1 Cys140 as an SNO site experimentally verified. Table 1 shows that four of the five computational programs predicted Cys148 in AtCOI1 as an SNO site. The X-ray structure of TIR1 in complex with a degron peptide for AUX/IAAs in the presence of auxin shows that Cys140 is located next to Ser139 residue suggested as part of TIR1 contact with AUX/IAA degron peptide (Supplementary Figure 3; Tan et al., 2007). Since Cys140 undergoes S-nitrosation, this modification may affect TIR1 contacts with AUX/IAAs. Coincidently, the tir1 C140A mutant protein is impaired in its interaction with IAA3 (Terrile et al., 2012). However, COI1 Cys148 putative S-nitrosated residue is not associated with COI1 interaction with the transcriptional repressors JAZ proteins but is surrounded by several residues associated with COI1-InsP5 interaction (Supplementary Figure 3; Sheard et al., 2010). COI1 and TIR1 crystal structures described the presence of inositol polyphosphates (InsP5 and InsP6, respectively) in their structural core, which act as cofactors for TIR1 and COI1 function (Sheard et al., 2010). A JAs-mediated induction of inositol pyrophosphate InsP8, which binds to COI1-JAZ co-receptor, was described as part of plant defense response against necrotrophic fungi and herbivorous insects in *A. thaliana* (Laha et al., 2015). Much more recently, it was demonstrated in animal systems that InsP6 and InsP7, in association with IP5K and IP6K enzymes, participate in the assembly of cullin-RING E3 ubiquitin ligase complexes (Zhang and Rao, 2019). In this context, COI1 putative S-nitrosation could affect COI1 activity by modifying inositol polyphosphates binding. In addition, other putative targets of S-nitrosation were detected (Cys60, Cys435, and Cys581 in four of five predictors; Cys406 in three of five predictors; Table 1). Cys406 residue is in the proximity of amino acids associated with COI1-JA-Ile and COI1-JAZ degron contact constituting an interesting Cys for redox regulation with impact on JA signaling (Supplementary Figure 3). Interestingly, at a biochemical and physiological level, COI1 proteins with point mutations in Lys81, Arg121, and Arg409 residues showed decreased interaction with JAZ protein and displayed a reduced capability to rescue JA-mediated root growth inhibition or silique development in *A. thaliana coi1* mutants (Mosblech et al., 2011). Those amino acids are less than 6Å distance from putative S-nitrosylated Cys60, Cys148, and Cys406, respectively (Supplementary Figure 6). Basic (Arg, His, Lys) residues surrounding the reactive thiol Cys sites are, in many S-nitrosated proteins, essential to favor the formation of the SNO adduct through electrostatic interactions (Kovacs and Lindermayr, 2013). These data support Cys60, Cys148, and Cys406 as good candidates for NO-mediated regulation by S-nitrosation. Similar to NO, hydrogen sulfide (H_2_S) gasotransmitter mechanism of action includes a redox posttranslational modification of Cys residues called persulfidation (Aroca et al., 2018). In the last years, proteomic approaches allowed the identification of a growing number of persulfidated proteins (Aroca et al., 2015; Aroca et al., 2017). There exist several proteins in plants where more than one redox posttranslational modification, including S-nitrosation, S-glutathionylation, and persulfidation, have been described (Yun et al., 2011; Iglesias et al., 2018; Shen et al., 2020). In this context, COI1 was found persulfidated in plants treated with NaHS as a cytosol H_2_S donor confirming it is a redox-regulated target protein (Aroca et al., 2017). The variety of thiol redox molecular switches of one or more Cys residues in a single protein, with similar or distinct functions including changes in stability, localization, or interaction with partners may allow versatile and rapid emerging regulatory process.

**TABLE 1:** Computational prediction of S-nitrosation sites from AtCOI1 protein using GPS-SNO 1.0, iSNO-PseAAC, iSNO-AAPair, DeepNitro, and pCysMod software.

Protein	Predicted SNO site position	Software	Sequence
	60	GPS-SNO (low threshold), iSNO-PreAAC, iSNO-AAPair, pCysMod (high threshold)	EHVTMAL**C**YTATPDR
COI1	148	GPS-SNO (low threshold), iSNO-PreAAC, iSNO-AAPair, DeepNitro (medium threshold)	ETLKLDK**C**SGFTTDG
	435	GPS-SNO (low threshold), iSNO-PreAAC, DeepNitro (high threshold), pCysMod (high threshold)	VRSLLIG**C**KKLRRFA
	531	GPS-SNO (low threshold), iSNO-PreAAC, iSNO-AAPair, DeepNitro (high threshold), pCysMod (high threshold)	LAGQRTD**C**PTTVRVL
TIR1	140	GPS-SNO (low threshold), iSNO-PreAAC, iSNO-AAPair, pCysMod (high threshold)	KVLVLSS**C**EGFSTDG

*Cys residues from AtCOI1 predicted in four or more predictors are shown. TIR1 Cys140 as reference for SNO site experimentally confirmed by the Biotin Switch method (Terrile et al., 2012) and MS (this work) is included. C in bold: matched Cys residue.*

### Nitric Oxide-Mediated Regulation of Jasmonate Signaling Pathway

**FIGURE 5:**
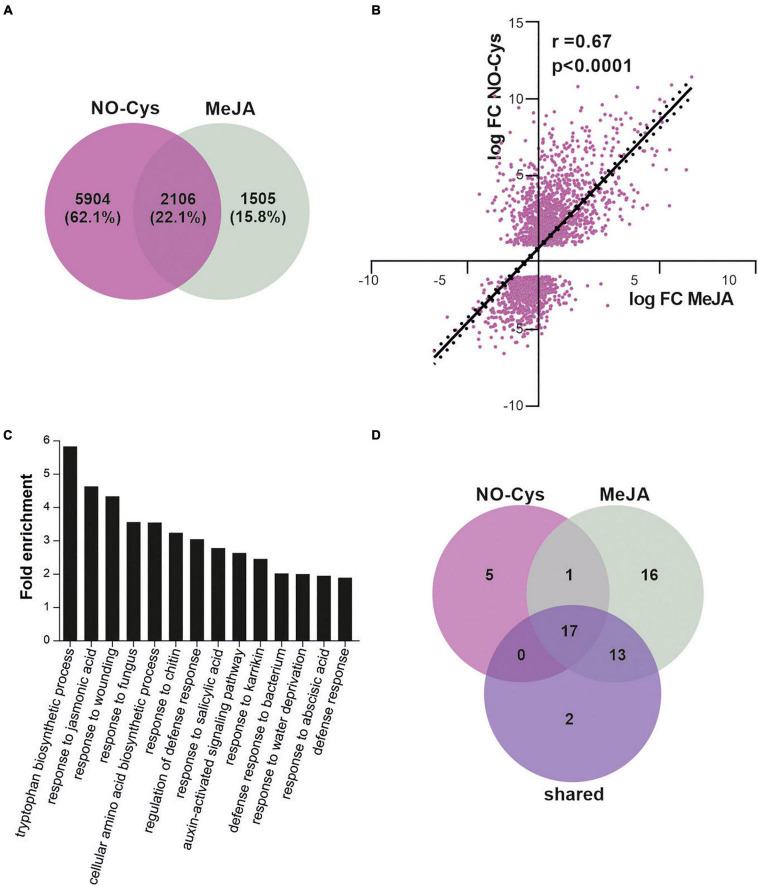
NO regulation of SCF^COI1^-mediated JA response gene expression. **(A)** Differentially expressed genes (DEGs) upon 6 h of NO-Cys treatment (purple) or 6 h of MeJA treatment (grey) in 3 weeks-old *A. thaliana* plants compared to control conditions. Raw data from Hussain et al. (2016) were downloaded and analyzed as described in section “Materials and Methods.” Only genes with absolute logFC > 1 and *p*-value < 0.0001 were selected as DEG. DEGs upon MeJA were selected from Hickman et al. (2017). **(B)** Correlation of change in expression levels for shared genes in **(A)** (2106 genes in total). Values are logFC after 6 h of treatment with NO-Cys or MeJA. Black line represents the linear regression line (*Y* = 1.112*X + 0.7580); *r* and *p*-value are indicated. **(C)** Gene Ontology (GO) term enrichment analysis of DEG upon MeJA and NO-Cys treatment. **(D)** Venn diagram displaying the number of overlapping GO terms highly enriched for NO-Cys, MeJA, and shared DEG.

Our results suggest that NO could affect the JAs signaling pathway by the putative S-nitrosation of the COI1 co-receptor and the demonstrated redox-modification of ASK1 scaffold protein resulting in a positive impact on SCF^COI1^ assembly required for COI1 stability. Then, we explore the NO effect of JAs signaling regulation at a genome-wide level by comparing the impact on the transcriptome of plants treated with agents that induce S-nitrosation to the transcriptome of plants treated with the volatile methyl ester JA (MeJA). We analyzed public RNAseq raw data from one experiment in which plants were treated 6 h with NO-Cys (Hussain et al., 2016). We found a clear impact on mRNA expression as 8,010 genes were affected in their transcription after the addition of the NO donor (Figure 5A). A set of 3,611 genes has been identified to be differentially expressed by MeJA (Hickman et al., 2017; Figure 5A). So far, the effect of treating plants with NO as well as MeJA resulted in a big impact on the transcriptome. Interestingly, from these MeJA-responsive genes, ∼58% (2,106 genes; Supplementary Table 2) are shared with NO-Cys treatments (Figure 5A; *p* = 3 × 10^–13^, hypergeometric test), suggesting that transcriptomic changes in these two experiments are much alike. Most importantly, the common genes show a positive correlation in expression, indicating that both signals regulate this subset of genes similarly (Figure 5B). These observations support our hypothesis on NO-mediated activation of the SCF^COI1^ signaling pathway. Moreover, functional classification of NO-Cys responsive genes by Hussain et al. (2016) identified hormone metabolism including JAs-signaling as a GO enriched term. To further analyze NO-JAs interaction and biological significance at a transcriptional level, the genes mutually regulated by NO-Cys and Me-JA treatments were tested for overrepresented functional categories using GO term enrichment analysis. Figure 5C shows functional categories associated with JAs responses and immunity significantly represented, including broad annotation categories as response to fungus, bacteria, chitin, wounding, and salicylic acid. However, DEG commonly regulated by NO-Cys and MeJA are specifically enriched in tryptophan (Trp) biosynthetic pathway. Effective defense responses of *A. thaliana* plants against filamentous pathogens have been associated with the JAs-mediated biosynthesis of Trp-derived metabolites as indole glucosinolates (Dombrecht et al., 2007; Liu et al., 2010; Pastorczyk et al., 2020). Indeed, NO-Cys seems to have a relevant impact on the transcriptional regulation of secondary metabolite biosynthetic processes including glucosinolates, flavonoids, tocopherol, carotenoids, and terpenoids during plant immune responses against fungal pathogens (Hussain et al., 2016; Mur et al., 2017). Although accumulating data suggest that NO production during plant-pathogen interactions contributes to the reprogramming of plant immune-response genes (Kolbert et al., 2019), the crosstalk between NO and JAs particularly in the induction of second metabolite biosynthesis has not been explored yet. In addition, categories including response to karrikin, water deprivation, and abscisic acid suggest possible participation of NO and JA in the transcriptional modulation of abiotic stress tolerance and germination processes (Wang et al., 2018). Surprisingly, the Venn diagram in Figure 5D shows a strong overlapping of GO term enriched categories between MeJA and NO-Cys, at least at this plant stage and time of exposure to both pharmacological treatments, suggesting that JA and NO trigger very similar transcriptomic reprogramming over the same molecular processes.

NO crosstalk with several phytohormones has been associated and extensively studied in plant development and abiotic stress responses (Sanz et al., 2015; Verma et al., 2020; Zhou et al., 2021). However, NO-mediated regulation of JA metabolism was circumscribed to NO-activation of JA biosynthesis genes (Mur et al., 2013). Table 2 shows that there exist diverse physiological processes where NO and JA signalings are mutually regulated. This work highlights a molecular framework for NO modulation of SCF^COI1^ complex with potential impact on physiological responses regulated by JAs during development and responses to stress.

**TABLE 2:** Crosstalk between JAs signaling and NO in diverse physiological processes.

Hormone	Physiological processes	JA signaling component identified	Function	Specie	References
MeJA	Stomatal closure	COI1	Induced NO and ROS production. Activated Ca^2+^ permeable cation channels and S-type anion channels.	*A. thaliana*	Munemasa et al., 2007
JA	Stomatal closure	ND	Induced NO production.	*Vicia faba*	Liu et al., 2005
JA	Primary root elongation and lateral root development	COI1/MYC2/JAZ1/JAZ10	Induced NO accumulation and expression of downstream JA repressors JAZ1 y JAZ10	*A. thaliana*	Barrera-Ortiz et al., 2018
JA	Wounding response	ND	Induced NO production	*A. thaliana*	Huang et al., 2004
JA	Basal defense against root-knot nematode, *Meloidogyne incognita*	COI1	NO-mediated JA-induced root-knot nematode resistance	*Solanum lycopersicum*	Zhou et al., 2015
	Elicitor-induced responses	ND	NO-mediated elicitor-induced hypericin production through a JA-dependent signaling pathway	*Hypericum perforatum*	Xu et al., 2005
MeJA	Induced defense responses	ND	Induced NO production and secondary metabolite activities	*Taxus chinensis*	Wang and Wu, 2005
MeJA	Chilling injury in postharvest fruit	ND	Induced NO-mediated postharvest chilling tolerance	*Cucumis sativus*	Liu et al., 2016
–	Induced resistance against *Botrytis cinerea*	COI1-JAZ1	eATP activated JA signaling via NO, maximizing defense responses. eATP increased COI1-JAZ1 protein-protein interaction	*A. thaliana*	Tripathi and Tanaka, 2018; Tripathi et al., 2018
MeJA	Response to *B. cinerea* in postharvest fruit	ND	Induced NO production and enhanced the resistance to *B. cinerea* by elevating defense-related enzymes and the phenylpropanoid pathway	*Vaccinium ashei*	Wang et al., 2020

## Conclusion

SCF E3 ubiquitin ligases constitute essential components of hormone metabolism and sensing. In this work, we provide evidence on NO regulation of SCF^TIR1^ and SCF^COI1^ complexes impacting the activation of auxin and JAs signaling pathways, associated with diverse physiological responses in plants. However, since ASK1 is part of diverse SCF complexes, S-nitrosation of ASK1 could potentially be implicated in the homeostasis of virtually every growth regulator, which could be associated, at least in part, with the versatile and broad action of NO as a second messenger in plant biology.

## Data Availability Statement

The datasets presented in this study can be found in online repositories. The names of the repository/repositories and accession number(s) can be found below: ProteomeXchange with identifier PXD030054.

## Author Contributions

MJI, MCT, and CAC conceived the project and designed the analysis. MJI, MCT, AM-R, DFF, LIACV, JLM, and ME contributed to experimental design, setup, and analysis tools. MJI, MCT, NMT, SLC, EM-L, NS-L, AI-Á, and AM-R performed experimental work and analyzed the data. MJI, MCT, and DFF wrote the manuscript. CAC and LIACV assisted with critical reading. All authors had a direct and intellectual contribution to the work, read and approved the final manuscript.

## Conflict of Interest

LIACV was employed by company KWS Gateway Research Center, LLC. The remaining authors declare that the research was conducted in the absence of any commercial or financial relationships that could be construed as a potential conflict of interest.

## Publisher’s Note

All claims expressed in this article are solely those of the authors and do not necessarily represent those of their affiliated organizations, or those of the publisher, the editors and the reviewers. Any product that may be evaluated in this article, or claim that may be made by its manufacturer, is not guaranteed or endorsed by the publisher.

## Footnotes

1
http://sno.biocuckoo.org/

2
http://app.aporc.org/iSNO-PseAAC

3
http://app.aporc.org/iSNO-AAPair

4
http://deepnitro.renlab.org

5
http://pcysmod.omicsbio.info./

## Supplementary Material

Supplementary Figure 1 MS/MS spectrum of TIR1 tryptic peptide including carbamidomethylated Cys140 peptide. TIR1 was treated with GSNO and subjected to a redox switch, substituting reversibly oxidized Cys to carbamidomethylation.

Supplementary Figure 2 Pull-down reactions were performed using *in vitro* synthesized TIR1-myc and recombinant GST-ASK1 or GST proteins. Interacting TIR1 protein was detected using an anti-myc antibody (upper panel). Coomassie blue-stained GST–ASK1/GST was used as a loading control (lower panel).

Supplementary Figure 3 Sequence alignment of *A. thaliana* TIR1, AFB1-5, and COI1. Critical ligand-, InsP6/phosphate-, and substrate-contacting residues are indicated by colored dots as described in the key.

Supplementary Figure 4 Cartoon representation of the ASK1-TIR1 crystal structure. A detailed view of the interface of interaction ASK1-TIR1 including the distance between selected residues is shown. ASK1 and TIR1 are represented in magenta and light blue, respectively. Cys118 ASK1 is colored in yellow; His16 and Val17 TIR1 are colored in green. Crystallographic data were obtained from Protein Data Bank, 2p1q.

Supplementary Figure 5 Alignment of selected COI1 orthologs showing the conservation in Cys487. Color code: dark gray: 100% similarity; medium gray: 80–100% similarity; light gray: 60–80% similarity; white: less than 60% similar.

Supplementary Figure 6 Distances between potentially S-nitrosable Cys and key residues in COI1. **(A)** Distances between Cys60 (red) and Lys81 (yellow). **(B)** Distances between Cys148 (red) and Arg121 (yellow). **(C)** Distances between Cys406 (red) and Arg409 (yellow). The distances between atoms are measured in Å.
