# Supplementary material for: S-Nitrosation of E3 Ubiquitin Ligase Complex Components Regulates Hormonal Signalings in Arabidopsis
Source: Front Plant Sci. 2022 Feb 4;12:794582. doi: 10.3389/fpls.2021.794582 (PMC8854210; doi:10.3389/fpls.2021.794582)
Supplement: Supplementary file 1 [file Table_1.DOCX]

**Supplementary Table S1. List of primers used for qPCR.**

| **Name** | **Locus** | **Primers** |
| --- | --- | --- |
| NbVSP1 | Niben101Scf34114g00003 | Fw 5’-GCAAGGATGTTTGGGTCTTTG -3’  Rv 5’-ACTGCTGGAGCTTTCCCTTCT-3’ |
| NbMYC2 | Niben101Scf06822g04004.1 | Fw 5’-GTGGTGAAGGAAGCGGATAGT-3’  Rv 5’-TTCTCCCTCCTTTGCCTCTCT-3’ |
| NbASA1 | Niben101Scf06493g00022.1 | Fw 5’-GAGGACGAGGAGAAGGAAGGA-3’  Rv 5’-CACGAGCATTTCCAGAGCTTC-3’ |
| NbPR4 | X60281 | Fw 5’-GGCCAAGATTCCTGTGGTAGAT-3’  Rv 5’-CACTGTTGTTTGAGTTCCTGTTCCT-3’ |
| NbEF-1α | [Niben101Scf12941g01003.1](https://solgenomics.net/tools/blast/show_match_seq.pl?blast_db_id=266;id=Niben101Scf12941g01003.1;hilite_coords=160-1487) | Fw5´-TGAGATGCACCACGAAGCTC-3´  Rv 5´-CCAACATTGTCACCAGGAAGTG-3´ |
|  |  |  |
