# Supplementary figures and images for: S-Nitrosation of E3 Ubiquitin Ligase Complex Components Regulates Hormonal Signalings in Arabidopsis
Source: Front Plant Sci. 2022 Feb 4;12:794582. doi: 10.3389/fpls.2021.794582 (PMC8854210; doi:10.3389/fpls.2021.794582)

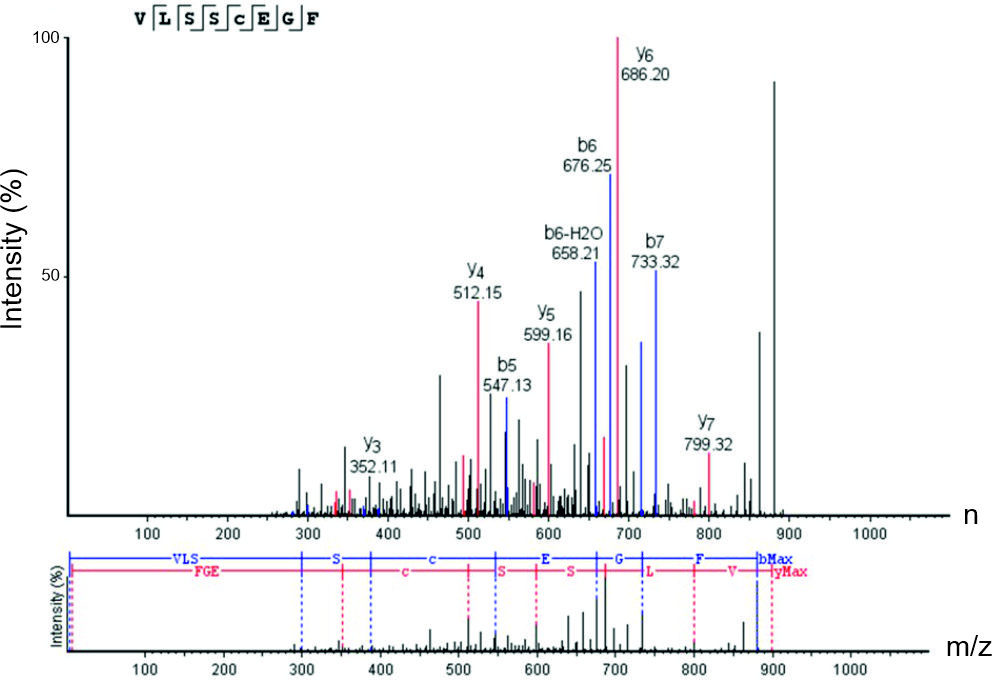

Supplement: Supplementary Figure 1 — MS/MS spectrum of TIR1 tryptic peptide including carbamidomethylated Cys140 peptide. TIR1 was treated with GSNO and subjected to a redox switch, substituting reversibly oxidized Cys to carbamidomethylation. [file Image_1.JPEG]

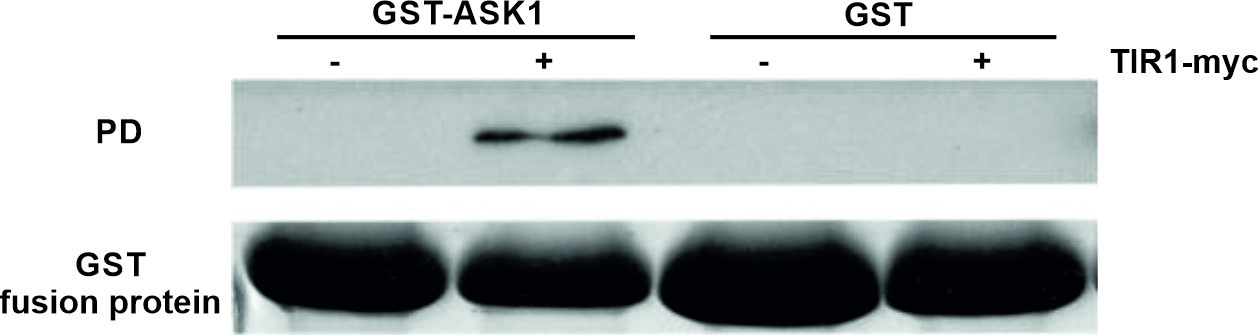

Supplement: Supplementary Figure 2 — Pull-down reactions were performed using in vitro synthesized TIR1-myc and recombinant GST-ASK1 or GST proteins. Interacting TIR1 protein was detected using an anti-myc antibody (upper panel). Coomassie blue-stained GST–ASK1/GST was used as a loading control (lower panel). [file Image_2.JPEG]

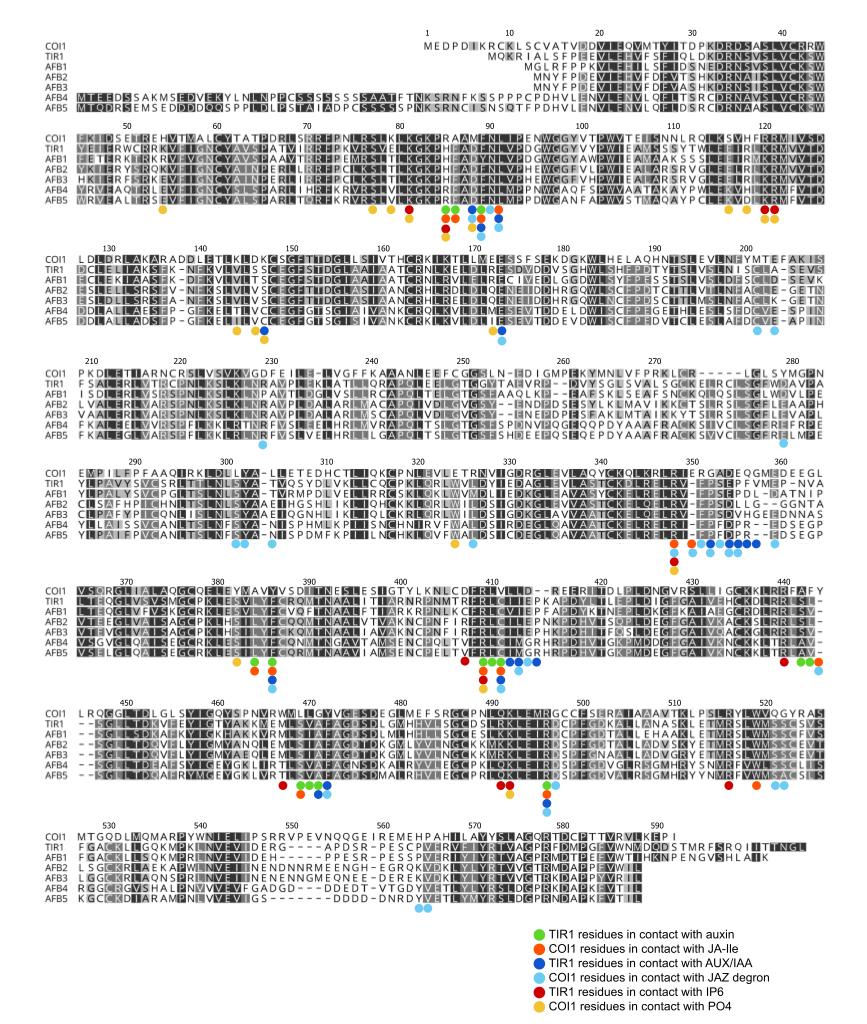

Supplement: Supplementary Figure 3 — Sequence alignment of A. thaliana TIR1, AFB1-5, and COI1. Critical ligand-, InsP6/phosphate-, and substrate-contacting residues are indicated by colored dots as described in the key. [file Image_3.PNG]

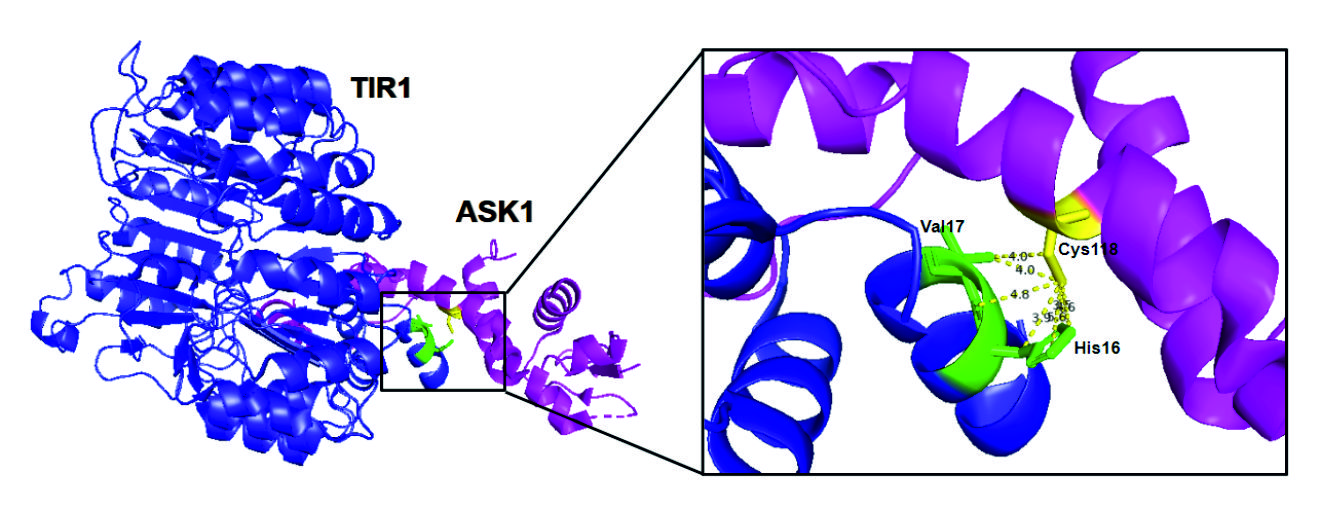

Supplement: Supplementary Figure 4 — Cartoon representation of the ASK1-TIR1 crystal structure. A detailed view of the interface of interaction ASK1-TIR1 including the distance between selected residues is shown. ASK1 and TIR1 are represented in magenta and light blue, respectively. Cys118 ASK1 is colored in yellow; His16 and Val17 TIR1 are colored in green. Crystallographic data were obtained from Protein Data Bank, 2p1q. [file Image_4.JPEG]

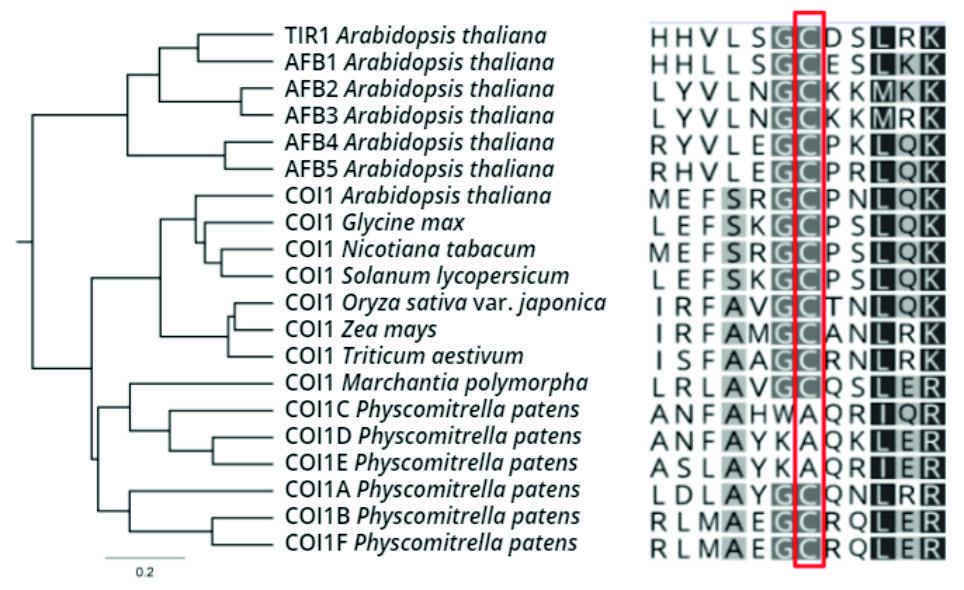

Supplement: Supplementary Figure 5 — Alignment of selected COI1 orthologs showing the conservation in Cys487. Color code: dark gray: 100% similarity; medium gray: 80–100% similarity; light gray: 60–80% similarity; white: less than 60% similar. [file Image_5.JPEG]

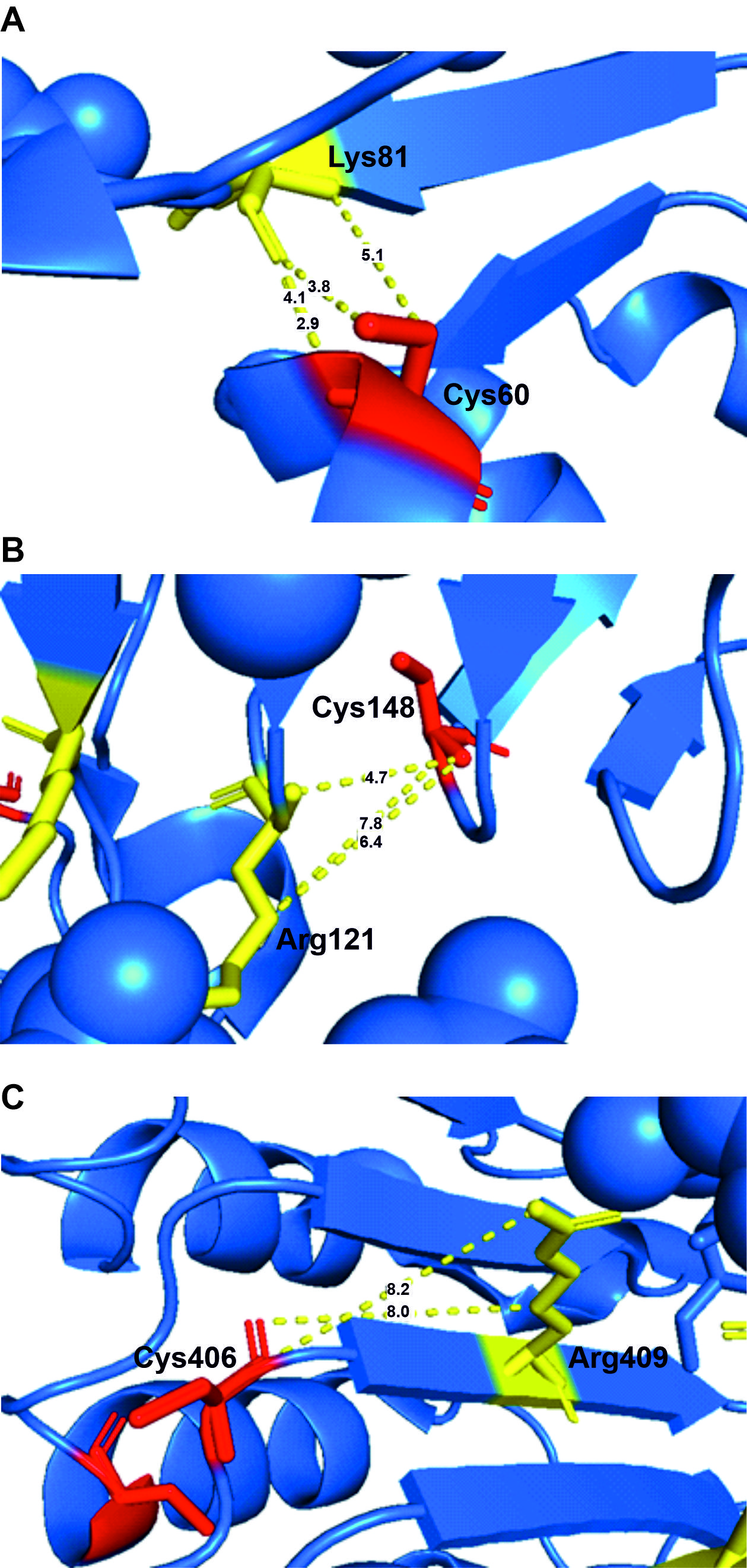

Supplement: Supplementary Figure 6 — Distances between potentially S-nitrosable Cys and key residues in COI1. (A) Distances between Cys60 (red) and Lys81 (yellow). (B) Distances between Cys148 (red) and Arg121 (yellow). (C) Distances between Cys406 (red) and Arg409 (yellow). The distances between atoms are measured in Å. [file Image_6.JPEG]
